# Sustainable Alternatives for the Development of Thermoset Composites with Low Environmental Impact

**DOI:** 10.3390/polym15132939

**Published:** 2023-07-04

**Authors:** Patricia Ares-Elejoste, Ruben Seoane-Rivero, Iñaki Gandarias, Aitziber Iturmendi, Koldo Gondra

**Affiliations:** 1 GAIKER Technology Centre, Basque Research and Technology Alliance (BRTA), Parque Tecnológico de Bizkaia, Edificio 202, 48170 Zamudio, Spain; ares@gaiker.es (P.A.-E.); seoane@gaiker.es (R.S.-R.);; 2 Chemical and Environmental Engineering Department, University of the Basque Country (UPV/EHU), Alameda Urquijo s/n, 48013 Bilbao, Spain

**Keywords:** sustainability, thermoset composites, bio-based, sustainable reinforcements, biocomposites applications

## Abstract

The current concerns of both society and the materials industries about the environmental impact of thermoset composites, as well as new legislation, have led the scientific sector to search for more sustainable alternatives to reduce the environmental impact of thermoset composites. Until now, to a large extent, sustainable reinforcements have been used to manufacture more sustainable composites and thus contribute to the reduction of pollutants. However, in recent years, new alternatives have been developed, such as thermosetting resins with bio-based content and/or systems such as recyclable amines and vitrimers that enable recycling/reuse. Throughout this review, some new bio-based thermoset systems as well as new recyclable systems and sustainable reinforcements are described, and a brief overview of the biocomposites market and its impact is shown. By way of conclusion, it should be noted that although significant improvements have been achieved, other alternatives ought to be researched.

## 1. Introduction

One of the most pressing problems in the 21st century is the continuing depletion of crude oil, i.e., the scarcity of petroleum-based products, and serious environmental problems such as pollution. This is why, for materials such as composites, increasing emphasis is being placed on research for renewable alternatives [1,2,3,4].

Thermosetting materials are mostly affected by this point, as they have the disadvantage of not being recyclable, which translates into an increase in waste landfills [1]. However, in recent years, thermosetting resins have been developed using renewable sources, which, despite not being recyclable, represent a significant improvement in terms of sustainability. On the other hand, in addition to these bio-based resins, the research sector has developed alternatives for the recycling of thermosets and vitrimers, which allows composites to be remolded using a heat source once cured.

In order to make these materials more sustainable, the use of more renewable reinforcements such as cores and natural fibers, which are used in various manufacturing processes to obtain products such as clothing, sports equipment, etc., has been increasing for several years [5,6,7,8], resulting in a production of approximately 30,000,000 tons/year. It should be noted that, in addition to the fact that these types of reinforcements have an excellent specific modulus and elongation at break, they contribute to improving the economic situation of farmers, as these fibers come from lignocellulosic products.

Throughout this review, several issues, such as bio-based resins (epoxy, benzoxazine, polyesther, etc.), sustainable reinforcements (cores, foams, natural fibers, etc.), recyclable routes for thermoset composites, and a brief analysis of the bio-based composites market, will be discussed.

## 2. Sustainable Thermoset Resins

The high interest in the development of sustainable thermosetting resins is mainly due to their high properties. Thanks to their ability to form crosslinked networks after curing, these resins present excellent chemical properties, good thermal and mechanical properties, and excellent stability [2]. Within this group of resins, the following could be found: epoxy resins, polyester, vinylester, and benzoxazine (phenolic). Nevertheless, to synthesize bio-based thermosetting resins, it should be taken into account that, due to the complex structures of biomass resources, it is not practical or efficient to use them directly. Thus, biomass needs to be transformed into more useful or simpler molecules called building blocks or intermediates [9]. In addition, among the possible sustainable alternatives, there are also furan resins, which can be obtained from agricultural by-products.

The following is a summary of the sustainable resins currently available on the market and the renewable sources from which they are derived. These have been developed through the exploitation of renewable feedstock, formula optimization, copolymerization, etc.

### 2.1. Furan Resin

As mentioned above, furan resin is obtained from agricultural by-products [10]. In particular, furfural is obtained from furan by catalytic hydrolysis of biomass. This is then transformed into furfuryl alcohol, and through condensation reactions and a Diels–Alder cycloaddition, furan resin is obtained.

The main advantage of this type of resin is that it is a good substitute for phenolic resins, which are in demand because of their good thermal stability, mechanical properties, and durability.

Table 1 below summarizes the advantages and disadvantages of these resins.

As mentioned above, the thermal stability of these resins is one of the most important factors. In fact, according to the research carried out by [11], this kind of resin could be used in the manufacturing of materials for the railway sector. Additionally, they are used in the foundry industry as sand binders for the manufacture of molds.

### 2.2. Bio-Based Benzoxazine Resin 

**Figure 1:**
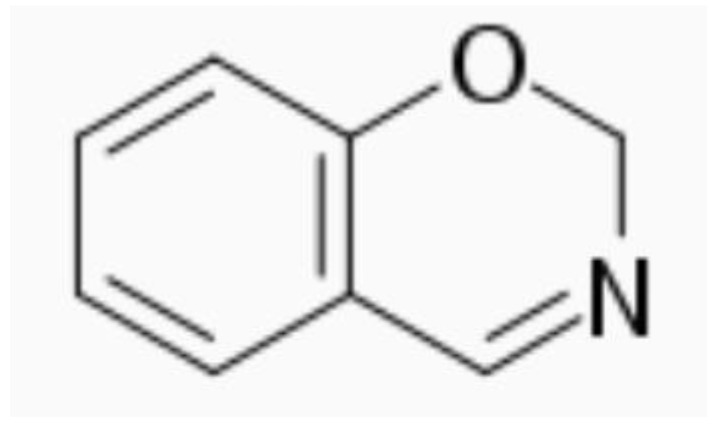
Benzoxazine structure.

Benzoxazine resin (Figure 1) is a type of thermosetting phenolic resin that has been gaining interest in recent years due to its many advantages, including low water absorption, good thermal stability, zero shrinkage, a high char yield, and no by-products during the curing reaction [2]. In terms of sustainability, these resins are good candidates, as they are flexible in their molecular design, which allows their synthesis using several types of phenols and amines, the components of which can be obtained from natural sources. In fact, thanks to the wide variety of sources from which these products can be obtained, it is possible to synthesize benzoxazine resins with properties even superior to those obtained from petroleum.

In fact, if a comparison is made between amines and phenols, in nature it is more common to find different types of phenols of biological origin that contain different substituents that allow the development of polybenzoxazine materials with different properties and characteristics.

One of the main natural sources of aromatic substances is lignin, from which compounds such as vanillin, eugenol, and guaiacol can be obtained and used for the synthesis of benzoxazine.

Some characteristics of these compounds are shown below:

- Guaiacol: a compound with a simple structure for which a copolymerization reaction is necessary in order to obtain better performance.
- Vanillin: a compound whose aldehyde group could remain intact during the synthesis of the benzoxazine monomers.
- Eugenol: it has both an allyl group and a phenolic hydroxyl group, which is how bio-based bisphenol can be obtained.

In addition to the compounds aforementioned, these types of resins can also be obtained from coumarin, sesamol, and arbutin.

With regard to the amines that can be used in development, the most widely used is furfurylamine, as it improves the hydrogen bonding system. Nevertheless, it should be noted that compared to phenols, there are not as many sustainable varieties, which makes it difficult to obtain resins with different properties.

Finally, other lignin-derived acids can also be used, such as ferulic acid, phloretic acid, and p-coumaric acid, whose carboxylic acid groups can lower the benzoxazine ring-opening temperature to around 130 °C.

### 2.3. Bio-Based Epoxy Resin

Among the bio-based resins, epoxy resins are currently the most developed. These resins have great advantages such as low shrinkage, easy moldability, good adhesion, and high resistance to weathering, which makes them very attractive for the numerous applications in various sectors. Most of these resins (nearly 90%) are developed through the use of epichlorohydrin and bisphenol A (in the presence of sodium hydroxide), the latter being a compound that is considerably harmful to both health and the environment.

**Figure 2:**
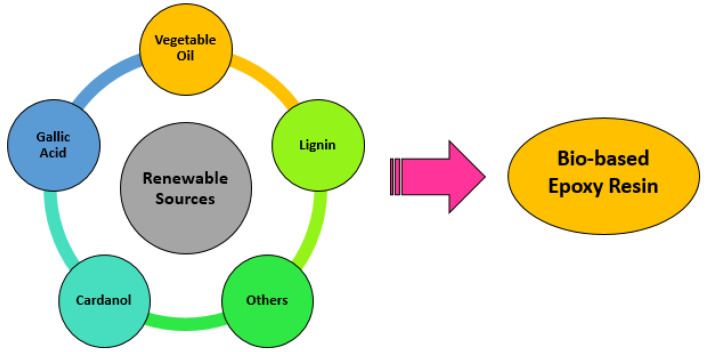
Renewable sources for the synthesis of bio-based epoxy resins.

For this reason, one of the key objectives has been the synthesis of these resins from natural elements. The methodology of the preparation of epoxy resins means that compounds of related renewable origin (Figure 2) have great potential for the synthesis of epoxy resins, which translates into a wide range of possibilities for obtaining this type of resin with bio-based content. Furthermore, it should be taken into account that, during the curing reaction, the epoxy group could be ring-opened by carboxyl, anhydride, or amine. It is therefore possible to design bio-based systems using a mixture of several renewable curing agents. To obtain bio-based epoxy resins, there are two types of routes: (i) direct reaction between apichlorohydrin and the bio-based compound (phenol or carboxylic acid), and (ii) the epoxidation of C-C double bonds into oxirane.

As can be seen in the picture above, there are different options for the synthesis of bio-based epoxy resins. However, considering that vegetable oils are the most suitable raw materials [3] and that they generally contain unsaturated double bonds, they are the best candidates to promote epoxidation reactions.

Due to the several renewable options through which these resins can be obtained, they can be separated into the following groups: (i) aromatic-containing bio-based epoxy resin; (ii) aliphatic bio-based epoxy resin; (iii) fully bio-based epoxy resin.

Within the aromatic bio-based epoxy resins, different sources for their synthesis, such as cardanol, lignin, or gallic acid, could be found.

Cardanol is a by-product extracted from cashew nut shells that contains a hydroxyl group, olefinic bonds in the alkyl chain, and an aromatic ring, making it an interesting substitute for bisphenol A in epoxy networks. However, it should be noted that, due to its inborn structural defects, the aliphatic chain present in this compound, causes the glass transition temperature values of the final materials to be lower than those obtained in resins with DGEBA (Bisphenol A diglycidyl ether). With respect to the mechanical properties, these are also inferior to those obtained with DGEBA. That is why copolymerization with other reactants is needed [2,9].

On the other hand, lignin is a very promising aromatic raw material of removable origin for the development of bio-based epoxy resins, as it is one of the most abundant biopolymers and also has phenolic hydroxyl groups that can react with epichlorohydrin. This compound is found in most plants (present in cell walls) and is generated in large quantities as a by-product of the paper industry, among others. Focusing on its structure, it is a complex and amorphous three-dimensional network whose content varies depending on the type of plant, accounting for around 15–40% of the dry weight of the lignocellulosic biomass. Due to its complicated structure and variation in molecular weight, its industrial applications are rather limited, as they tend to result in low yields. Therefore, fractionation or pre-treatment of lignin is currently being used to develop low polydispersity and more homogeneous starting materials.

With regard to the synthesis of bio-based epoxy resins, these compounds must also be modified prior to the epoxidation process, as lignins are not soluble and have low functionality. The most common processes for using lignin are summarized below:

- Direct mixing of lignin with epoxy resin;
- Modification of lignin by glycidylation;
- Modification of lignin derivatives and subsequent glycidylation.

According to the latest research by Liu et al. (2021), copolymerization of partially depolymerized lignin is one of the most promising methods. In addition, lignin derivatives are the most suitable for the synthesis of epoxy resins. Among them, vanillin, which has been mentioned above in the section on benzoxazine resin synthesis, stands out. Furthermore, apart from vanillin, another compound that stands out among others is eugenol, as it has two functional groups, the phenolic hydroxyl group and the allyl group, which can be modified to precursors with a variety of functionality for bio-based epoxy resins. In comparison to traditional BPA-based (Bisphenol A-based) epoxy resins, it should be noted that bio-based materials derived from lignin exhibit a higher limiting oxygen index (LOI), which can translate into a more effective material in terms of fire retardancy.

Finally, gallic acid is a compound derived from hydrolytic tannins, with three phenolic hydroxyl groups and a carboxylic group in its structure that can be used for synthesis.

Within the aliphatic group, vegetable oils (a mixture of esters derived from glycerol and unsaturated fatty acids) stand out as one of the main raw materials in the development of bio-based epoxy resins, which are obtained through the epoxidation reaction with molecular oxygen or through chemoenzymatic reactions. These include castor oil, soybean oil, microalgae oil, and residual vegetable oil, among others [2,9]. In general, these types of oils have been widely used for products such as cosmetics, lubricants, coatings, resins, etc.

In addition to oils, sucrose, or aliphatic polyols of biological origin, such as glycerol or sorbitol, are other valid alternatives.

Fully bio-based resins are considered to be those systems in which the epoxy precursor and hardener are from renewable sources. The curing agents used for the cross-linking of epoxy resins are generally polyamines and carboxylic compounds. Therefore, in order to be able to obtain a 100% bio-based system, one of the most attractive alternatives is to give vegetable oils the appropriate functionality so that they can also act as hardening agents in the system. As an example, lignin-derived curing agents can be prepared using either of two methods: (i) the reaction of lignin with ozone in the presence of NaOH (which provides the lignin with unsaturated carboxyl groups); or (ii) modified lignin together with, for example, anhydrides.

### 2.4. Bio-Based Unsaturated Polyester Resin

Polyester resins (Ups) are obtained from the condensation of a saturated dicarboxylic acid or its anhydride as well as an unsaturated dicarboxylic acid with dialcohols such as ethylene glycol or propylene glycol. Once the condensation reaction has taken place, the carbon-carbon double bonds polymerize to form a cross-linked network through the addition of free radicals [9,12]. 

These resins have many advantages, such as high workability, good mechanical, electrical, and chemical properties, and a low cost compared to epoxy resins.

In addition, in recent years, there has been increasing interest in the synthesis of these resins using removable raw materials such as diacids and diols. For example, itaconic acid has a carbon-carbon double bond and two carboxyl groups, so it has a high similarity to maleic acid. It is therefore a very interesting substitute.

However, it should be noted that very few bio-based products contain at least one benzene ring together with two carboxyl groups or two hydroxyl groups with reactivity. The furan ring is the one that provides this type of resin with the necessary rigidity. Therefore, in order to obtain bio-based UP resins with good thermo-mechanical properties, itaconic acid is a good alternative. In addition to this, there are other alternatives such as resorcinol, although this requires prior treatment due to its low nucleophilicity, or eugenol, which has a phenolic fraction and an allyl group.

In Table 2, some natural sources are shown in order to summarize the bio-based elements from which thermosetting resins and other substances can be obtained.

## 3. Sustainable Reinforcements

### 3.1. Natural Fibers

Natural fibers have demonstrated their capabilities in load-bearing applications due to their strength and stiffness. Compared with traditional composites, natural fiber composites have attracted much attention in the industry due to their density and environmental friendliness (https://doi.org/10.1016/j.jobe.2020.101411 (accessed on 14 May 2023)). Natural fibers consist of many elongated fibrils of cellulose and lignin, which associate with hydrogen bonds to provide strength and inflexibility. Synthetic fibers show better mechanical and physical properties compared to natural fibers. The specific modulus and elongation at break are better in natural fibers than in synthetic ones, which is considered an important factor in polymer engineering composites [13].

**Figure 3:**
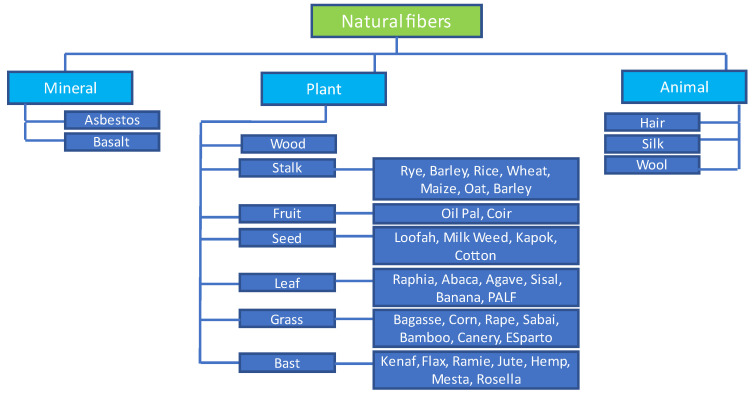
Classification of sustainable fibers.

Besides vegetable fibers, there are also different kinds of different animal fibers, such as those made of wool, silk, feathers, bird fibers, and animal hair, which are the most important resources. Straw fibers are collected from the husks and straws of crops such as wheat, rice, and barley. Natural fibers can be obtained in bundles from many parts of plants, such as bast stems, leaves, and seeds. Fiber classifications are shown in Figure 3.

#### 3.1.1. Natural Fiber-Polymer Composites

Natural fiber composites are materials made of a polymer matrix embedded with high-strength natural fibers such as flax, jute, oil palm, and kenaf [14,15,16]. One reason for their growing use is that natural fibers have a similar stiffness to glass fibers and higher strength [17]. Because of these characteristics and cheaper sources, these natural fibers offer advantageous strengths and stiffness at a lower cost [18].

There are some aspects that affect the characteristics and performance of these kinds of composites, such as moisture [19], the hydrophilic nature of natural fibers, and fiber loadings [20,21,22]. It is usually noticed that high fiber loadings provide good mechanical strength. Another variable to take into account is the chemical composition of natural fibers. Their structure is normally composed of cellulose, hemicellulose, lignin, and waxes, contributing to the characteristics of composites. Studies have been reported on the suitability, competitiveness, and capabilities of natural fibers with matrixes. Researchers have studied the compatibility of natural fibers and matrix by using various surface modification techniques and manufacturing processes. Moreover, processing techniques and parameters are other factors affecting composites’ characteristics. The selection of appropriate processing techniques provides the best characteristics for producing composites. Some studies have been done on the basis of comparative studies of natural fibers with particular polymers [23].

#### 3.1.2. Hybrid Composites

Natural fiber-reinforced hybrid composites are created in order to be environmentally friendly and meet the demands of industries looking for sustainability. Reinforcing two or more natural fibers into a single matrix develops a hybrid composite. There are many researchers [24,25] who have tried to select the best combination of natural fibers to achieve the best outcome for utilization and minimize the negative aspects. Combinations of different types of fibers in a single matrix can generate synergistic results and, therefore, highly valued hybrid biocomposites. Basically, three types of reinforcement methods have been incorporated: (1) a mixture of two types of short fibers before adding matrix [26], or (2) adding fibers into polymer alternatively layering a fiber mat or fabric and matrix [27]; (3) in the case of glass fiber-LC fiber composite systems, addition of nonwoven and woven fabric in both types of reinforcements [28,29].

### 3.2. Sustainable Cores

Composite sandwich structures are widely applied in the aerospace, marine, transportation [30,31,32,33], and construction industries. The sandwich structure consists of two (top and bottom) skins or facings bonded to a core material [34]. The main function of this core material is to give the composite sandwich structures high compressive and flexural strength, as well as greater rigidity, without significantly increasing the overall weight of the composite material.

**Figure 4:**
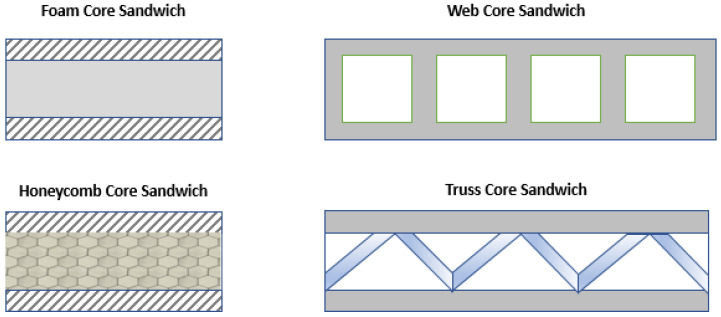
Sandwich construction configurations.

The core structure allows sandwich panels to be tailored to specific applications, where the most commonly used core structures are foams [35,36] or solid cores, honeycomb cores, truss cores, and web cores [37] (Figure 4). Polymer foam cores, for example, are widely used for car flooring, boat parts, and turbine blades due to their resistance to fatigue and temperature, good rigidity, and high strength [38,39,40,41]. On the other hand, honeycomb cores are suitable for aerospace [41], automotive, sports, and marine industries.

Thus, composite core materials have to meet the requirements of the application in which they will be placed; these requirements may lead to the fulfilment of the following properties: resistance to chemicals, moisture, corrosion, etc. For this reason, for its application, specific materials and configurations need to be selected.

Even though many composite core materials are well-established, increasing awareness of environmental issues has led to the use of new sustainable materials for sandwich structures [33]. Although there is still a need to search for and manufacture more environmentally friendly materials, there are currently some companies, such as EconCore, that have developed sustainable core materials based on natural materials. Nowadays, the sustainable core materials available are wood, Poly-Lactic Acid (PLA), and tannin, among others.

PLA is a biodegradable polymer obtained from renewable sources, such as sugar fermentation or starch-rich products, and has gained much attention in the last few years. Du et al. [42] developed a bio-based sandwich structure made of both skin and core materials from biofiber and PLA matrix. The findings indicated that the newly developed material met the automotive specification requirements for load floor flexural properties. Lascano et al. [43] have developed a highly sustainable sandwich structure made of a PLA honeycomb core and PLA/flax skin faces with balanced mechanical properties for medium-to-high technological applications. Furthermore, the TU/Ecomotive team from Eindhoven University of Technology [44] has proven the suitability of using PLA honeycomb core from EconCore to produce the chassis, body, and interior of the world’s first car made from biocomposites.

Balsa wood is widely used in the fabrication of wind turbine blades, boats, decks, small aircraft, etc. [45]. It is a light and natural material known to be good for thermal and acoustic insulation. Although the balsa core can be used as blocks or lumber, its substitution by thin veneer layers shows improved properties. Shir Mohammadi et al. [46], for example, demonstrated that laminated veneer lumber (LVL) balsa improved the toughness of a core material compared to solid balsa, although it is dependent on the lamination adhesive. Wu et al. [47] similarly confirmed the advantage of using veneered balsa wood in comparison to block material, since the first one reduces property scatter. Nowadays, veneer-based core materials are commercially available as Baltek^®^ VBC from 3A Composites. On the other hand, Gurit^®^ commercializes end-grain balsa wood core in a wide range of densities, thicknesses, formats, and finishes under the trade name Balsaflex™.

Cork is a truly lightweight material and a good electric, thermal, sound, and vibration insulator, in addition to being impermeable to gases or liquids [48]. When this material is used as a core in a sandwich structure, it offers a high stiffness-to-weight ratio, a high strength-to-weight ratio, thermal and acoustic insulation, etc. [49].

Sargianis et al. [50] showed that sandwich materials made of carbon fiber face sheets and cork agglomerate as a core material provided great performance in both acoustics and vibration. The authors claimed that these results could present a good solution in the aircraft and aerospace industries.

In a different study, Hoto et al. [51] developed a sustainable asymmetric sandwich material with basalt and flax natural fibers, an agglomerate cork panel as a core, and a bio-based epoxy resin as a matrix. The results showed good energy absorption behavior during the bending test and how the infiltration of the resin inside the core reduced the water absorption. Additionally, Torres et al. [52] presented the mechanical performance under tensile and flexural loads of this sandwich material composition. A longboard was manufactured as a demonstrator, and the results to failure showed acceptable performance for service conditions.

Amorim Cork Composites commercializes CORECORK materials made of cork granules agglomerated and free of plasticizers. The portfolio offers core materials for lightweight composite structures and products, as well as sandwich materials for residential and industrial applications. 

Last but not least, polymer foams derived from diverse bio-sources, such as tannin [53], starch [54], flax oil, etc., or recyclable processes, such as recycled PET foams [55], have been investigated or even commercialized. It is the case of ArmaFORM PET MC foam developed by Armacell, made from 100% post-consumer recycled PET, fully recyclable, and with improved compression strength and impact performance. 

## 4. Composite Recyclability

In recent years, there has been concern about the accumulation of waste composite materials due to the vast number of wind turbines, aircraft, and boats, for example, reaching their End-of-Life and consequently being decommissioned. Furthermore, if we think about the ban that countries can impose on composite landfilling in the coming years, as Germany did in 2009, the reuse and recyclability of composite materials has become necessary.

This section will not describe each recycling method available for composite materials (mechanical, thermal, and chemical recycling), but will present the most recent studies and developments on recyclable thermoset resins.

**Figure 5:**
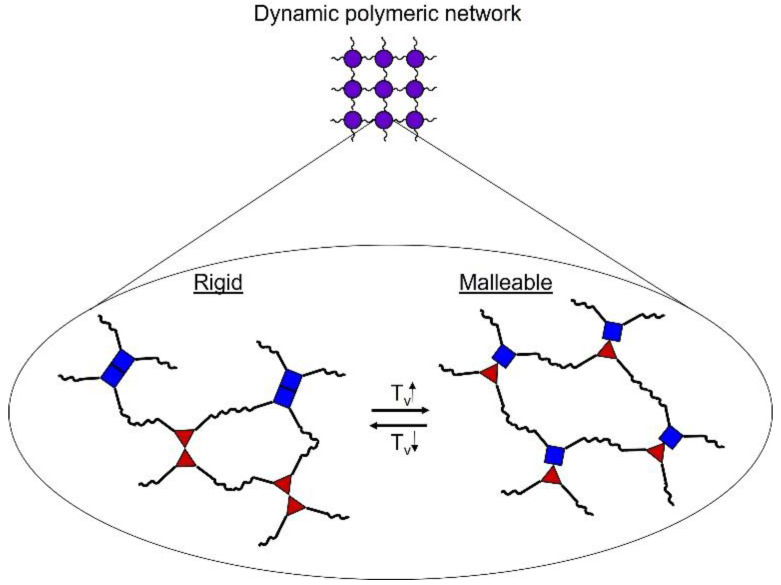
Schematic representation of the vitrimer system.

Liu et al. presented a fully recyclable phenolic-based resin using commercial novolac resin and toluene diisocyanate by forming a dynamic cross-linked network [56]. The advantage consisted of avoiding catalysts and exhibiting good recyclability with a high Tg value (up to 200 °C), in addition to almost equal mechanical strength even after five crushing and molding cycles. In this case, the presented reversibly exchangeable topology is comparable to that of vitrimers. Vitrimers are characterized by their ability to flow above their topology’s freezing transition temperature, Tv (the temperature at which the material changes from viscoelastic solid to viscoelastic liquid), while becoming rigid (like a thermoset) below it (Figure 5).

As can be seen in Figure 5, in vitrimers there is a dynamic polymerix network where, below the Tv, rigid bonds are obtained, so that the material is not malleable. On the other hand, when the material is subjected to high temperatures (above its Tv), these bonds can be exchanged with others in the network. In this way, the material can be thermoformed.

The dynamic networks present in vitrimer materials are based on exchangeable reactions, such as imine-amine exchange, transesterification, and disulfide exchange, among others. 

The first commercially available vitrimer material is based on imine-amine exchange, developed by Taynton et al. [57,58] under the name Vitrimax™ and commercialized by Mallinda [59]. They offer diverse Vitrimax™ materials with different properties (Tg values, for example) and oriented to different manufacturing processes. In order to understand the manufacturing method of this kind of material, He et al. [60] recently presented the network malleability, interfacial welding, and solvent-assisted recyclability of these materials following the previous work of Taynton et al. [57]. It demonstrated the recyclability process and the reusability of the recycled polyimine for the next group of composite manufacturing.

In different studies, disulfide-containing hardeners have been applied to develop reversible, self-healing, and malleable epoxy resins. It is the case of the work presented by Zhang et al. [61], in which 1,4,5-oxadithiepane-2,7-dione (DSAA) was designed and synthesized to be used as a hardener along with methylhexahydrophthalic anhydride (MHHPA) as a co-curing agent. The developed resin showed high mechanical strength and good thermal resistance (Tg of 113 °C), meeting the requirements for electronic packaging, in addition to good malleability and reversible self-healing ability. Preliminary tests about the recyclability process have also been presented, although it still needs to be improved. 

Since 2016, extensive work has been undertaken using aromatic disulfide bonds to crosslink epoxy resins and develop a new generation of fiber-reinforced thermoset composite materials with good (re)processability, reparability, and recyclability [62,63]. The processability/reshaping was proven and compared to a reference thermoset material, in which the newly developed material showed good processability in a hot press at 200 °C at 100 bar for 5 min. Furthermore, the mechanical recycling of such material was also presented, showing comparable mechanical properties to the reference material after the recycling process. Recently, the same group obtained an aero grade epoxy resin with high Tg values (175 °C) while being sustainable [64]. The solution to reaching higher Tg values comes from the idea of introducing some fraction of permanent crosslinks (up to 30%) in order not to lose the reprocessability of the material. 

**Figure 6:**
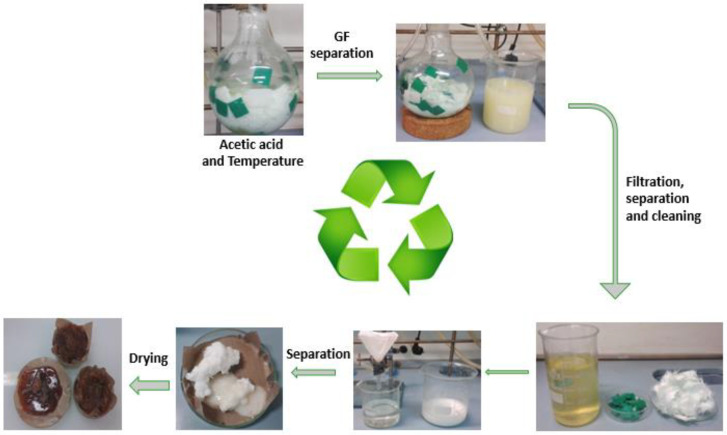
Recycling process of epoxy-based resin crosslinked with Recyclamine™.

Following the idea of recycling epoxy resins, it has been extensively demonstrated that it is possible to recycle epoxy-based composite materials to recover at least the reinforcements while keeping their properties. It is the case of the Recyclamine™ technology, originating from Connora Technologies and acquired by Aditya Birla in 2019. The recycling process is based on placing the composite in a dilute acetic acid solution at approximately 70–80 °C for 1 h. After this time, the reinforcements are separated, filtered, and cleaned to remove as much acetic acid as possible [65]. The recycled thermoplastic epoxy mixture is then neutralized with NaOH base, and after its precipitation, the mixture is filtered, rinsed, and dried (Figure 6). The approximated properties of the recovered epoxy-thermoplastic are provided in Table 3.

As can be seen in Table 3, if the recovered epoxy thermoplastic is compared to a conventional bio-based epoxy resin, properties such as elongation and Tg decrease considerably. However, with respect to modulus and tensile strength, we see that there is a slight decrease. Nevertheless, the properties are still good.

Thanks to the fully recyclable epoxy resin system, more sustainable composite materials have been developed. It is the case of the work presented by Cicala et al. [67], in which bio-based resins alongside hybrid flax/carbon fibers were used to manufacture hybrid and recyclable composites. Furthermore, the reprocessing possibilities of the recycled thermoplastic with both a microinjection molder and a single-screw extruder to produce filaments for fused deposition modeling (FDM) processing are described, showing good mechanical properties.

In a similar study, Ferrari et al. showed the possibility to recycle bio-based thermoset composites derived from waste flours [66]. This research opens the opportunity to recycle organic waste, minimizing the negative impacts on the environment.

In 2014, Arkema started the production of the liquid thermoplastic Elium^®^ resin. This resin is based on acrylic monomers, which have similar properties to thermosets but are easily recyclable. Its main advantages are the liquid state, which facilitates its processability like thermoset resins, the ability to polymerize at room temperature and harden more quickly, and the possibility of being easily recycled in contrast to thermoset resins [68]. In the investigations carried out by Gebhardt et al. [69], they used a matrix composed of virgin Elium^®^ and Elium^®^ mixed with different percentages of recycled Elium^®^ already polymerized in the form of granules. After a dissolution process and the manufacture of CFRP laminates, these were compared with those made from 100% virgin Elium^®^ and resulted in good mechanical properties. Furthermore, in order to recycle these materials, this type of resin can be both reprocessed and recycled by solvolysis with a common solvent.

Another recently developed alternative is the Akelite resin, patented by CSIC. This system is an acrylic resin with good mechanical properties and the ability to be thermoformed once cured, as well as being recyclable by immersion in acetone [70].

Among the thermoforming options, the epoxy systems from Cecence [71] also stand out, as they can be used in a wide range of sectors due to their properties. Specifically, there are three systems on the market: K_Chip, K_Plate, and K_Rod, which have considerably low molding temperatures as well as swirling of the cured part. 

Finally, one of the new developments that is not yet available on the market is the SWANCOR system known as “EzCiclo”, which is an epoxy resin with the capacity to be recycled by solvolysis with the solvent “Cleaver”, which allows the recycled resin resulting from the process to be reused.

## 5. Applications

Biocomposites applications in sectors such as the automotive sector have experienced exponential growth in Europe, as according to European regulations (EURO 6) in 2020, those cars that generate CO2 emissions above 95 g/km will receive a penalty [72]. Therefore, the use of biocomposites in this type of sector helps reduce these emissions. In fact, it should be noted that, in addition, the European Directive 2000/53/EC has established the objective of recycling 95% by weight of automobiles, creating a value chain with the use of biocomposites.

With regard to the different sectors that make use of this type of material, the following stand out [72]:

Tissue Engineering

This type of material is used in bone regeneration. Generally, biodegradable materials with good mechanical properties are used. In fact, porous nanomaterials have recently been developed for use in tissue engineering, as polymeric materials do not interfere with cell growth. 

Advanced Electronic Devices

Within the electronics sector, multifunctional biocomposites are used for the fabrication of innovative devices such as medical devices, displays, sensors, etc. A clear example of this are cellulose nanofibers, which are used in the manufacture of wiring, etc.

Automotive

Compared to conventional composites, biocomposites are lighter in weight. For this reason, they are used in the automotive sector, as they provide a reduction in CO_2_ emissions and significant fuel savings. Generally, these types of materials are used in vehicle interiors (dashboards, door panels, etc.), mainly due to their strength and the moisture absorption of natural fibers.

Other applications

In addition to the sectors aforementioned, biocomposites have started to be used in aircraft interior panels, wind blades, and some housing elements, such as flooring, roof panels, and door frames, among others.

### Biocomposites Market Size

**Figure 7:**
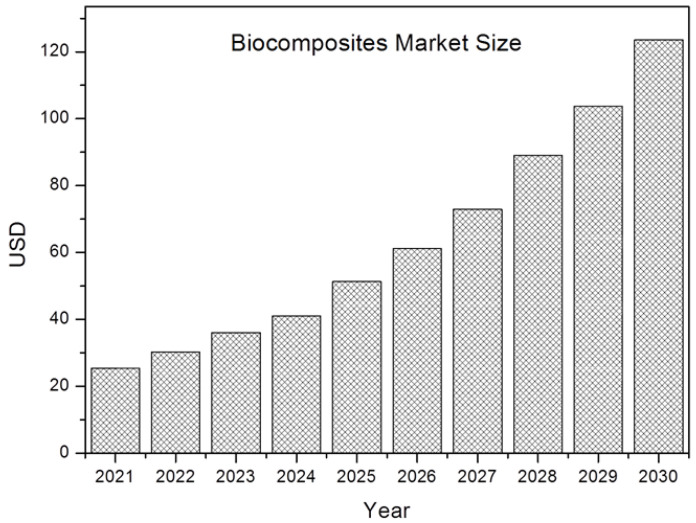
Biocomposites market development.

Currently, as discussed in Section 2.3 above, epoxy resins with a bio-based content of around 30% are available, which have a considerably lower environmental impact. Such systems and other types of bio-based resins are already being used in numerous applications together with natural reinforcements in various sectors such as aerospace, automotive, and electronics for secondary applications. Additionally, the use of biocomposites is expected to increase in the coming years. In fact, according to a study published by [72,73] during the period 2018–2030, the composites market was valued at USD 24.59 billion (Figure 7) in 2021 and is expected to increase at a CAGR (Compound Annual Growth Rate) of 16.1%.

In fact, developed countries such as Germany, the US, and Japan are increasingly focusing on the use of more sustainable products that promote recyclability. 

In order to use such materials in other, more novel applications, it is vital to identify the processing techniques and conditions, as well as the properties of the matrices, fibers, and their interfaces. Thus, for the development of biocomposites, it is essential to have prior knowledge of the constituent materials in order to achieve the properties demanded by each of the sectors and applications.

## 6. Conclusions

Throughout the review, sustainable alternatives to conventional thermoset systems have been analyzed. As has been observed, there are currently several alternatives to reduce the environmental impact of composite materials. However, it is still necessary to invest in research in order to obtain thermosetting resins with a higher bio-based content and, at the same time, provide similar properties to resins from fossil sources.

On the other hand, with respect to recyclable and/or thermoformable systems, these have very interesting properties, as most of them have similar properties to the initial ones once the recycling process has been carried out. However, other systems, such as Recyclamine, mentioned above, lead to the possibility of using the recycled material in a second life where the thermoplastic material has a place.

In conclusion, it is considered that the advances made in terms of sustainability do present an important improvement, but it is necessary to look for other alternatives in future actions that address the environmental problem in a more complete way.

## Figures and Tables

**Table 1:** Advantages and disadvantages of furan resins.

Advantages	Disadvantages
Derived from renewable resources	High variation of viscosity with temperature
Good chemical and thermal resistance	High shrinkage during curing
Free monomer content: ≥1%	H_2_O generation during polymerization
Good fire properties	
Similar performance to phenolic resins	

**Table 2:** Natural sources for the development of bio-based products.

Resource	Related Chemical	Functional Group	Bio-Based Products
Carbohydrate	Itaconic acid	Carboxyl; C-C double bond	Epoxy resin; Ups; Ups reactive diluents
	Furfuryl amine	Furan group	Benzoxazine and Epoxy resin
	Isosorbide	Alcohols; Diheterocycles	Ups; Epoxy and curing agent
Lignin	Vanillin	Aldehyde	Benzoxazine and Epoxy resin
	Eugenol	C-C double bond	Benzoxazine and Epoxy resin; Ups reactive diluents
	Guaiacol		
Vegetable Oils	Glyceride	Ester group; unsaturated aliphatic chain	Epoxy resin and curing agent; Ups; Ups reactive diluents
	Cardanol	unsaturated aliphatic chain	Benzoxazine; Epoxy resin

**Table 3:** Average of properties of the recovered epoxy-thermoplastic vs. conventional bio-based epoxy with Recyclamine™ [65,66].

	Recovered Epoxy-Thermoplastic	Conventional Bio-Based Epoxy
Properties	Value	Value
Glass transition temperature (Tg)	40–60 °C	105
Melting temperature (Tm)	120–140 °C	-
Tensile modulus	2.4 GPa	2.8–3.2 GPa
Tensile strength	57 MPa	75 MPa
Elongation	45%	5–8%
Shore D hardness	80	-

## Data Availability

The data reported in this study are available upon request from the corresponding author.

## References

[1] Dinu R., Montes S., Orange F., Mija A. (2021). Reprocessable humins thermosets and composites. Compos. Sci. Technol..

[2] Liu J., Zhang L., Shun W., Dai J., Peng Y., Liu X. (2021). Recent development on bio-based thermosetting resins. J. Polym. Sci..

[3] Kousaalya A.B., Beyene S.D., Ayalew B., Pilla S. (2019). Epoxidation Kinetics of High-Linolenic Triglyceride Catalyzed by Solid Acidic-Ion Exchange Resin. Sci. Rep..

[4] Pearson R.A., Craver C.D., Carraher C.E. (2000). Thermosetting plastics. Applied Polymer Science: 21st Century.

[5] Watson K.J., Wiedemann S.G. (2019). Review of Methodological Choices in LCA-Based Textile and Apparel Rating Tools: Key Issues and Recommendations Relating to Assessment of Fabrics Made From Natural Fibre Types. Sustainability.

[6] Vanitha R., Kavitha C. (2021). Development of natural cellulose fiber and its food packaging application. Mater. Today Proc..

[7] Fortunati E., Verma D., Luzi F., Torre L. (2021). Natural Fibre Based Biopolymer Formulations with Potential Applications in Biomedical and Packaging Sector. Mini-Rev. Org. Chem..

[8] Mashkour M., Mashkour M. (2021). A Simple and Scalable Approach for Fabricating High-Performance Superparamagnetic Natural Cellulose Fibers and Papers. Carbohydr. Polym..

[9] Liu J., Wang S., Peng Y., Zhu J., Zhao W., Liu X. (2021). Advances in sustainable thermosetting resins: From renewable feedstock to high performance and recyclability. Prog. Polym. Sci..

[10] McKillip W. (1989). Adhesives from Renewable Resources.

[11] Elejoste P.A., Allue A., Ballestero J., Neira S., Gómez-Alonso J.L., Gondra K. (2022). Development and Characterisation of Sustainable Prepregs with Improved Fire Behaviour Based on Furan Resin and Basalt Fibre Reinforcement. Polymers.

[12] Ma S., Li T., Liu X., Zhu J. (2016). Research progress on bio-based thermosetting resins. Polym. Int..

[13] Kabir M., Wang H., Lau K., Cardona F. (2012). Chemical treatments on plant-based natural fibre reinforced polymer composites: An overview. Compos. Part B Eng..

[14] Chu L., Shi J., Yu H., de Cursi E.S. (2019). Uncertainty propagation in moisture absorption of flax/glass fiber reinforced hybrid composites. Mater. Res. Express.

[15] Laftah W.A., Majid R.A. (2019). Development of bio-composite film based on high density polyethylene and oil palm mesocarp fibre. SN Appl. Sci..

[16] Feng N.L., Malingam S.D., Ping C.W., Razali N. (2020). Mechanical properties and water absorption of kenaf/pineapple leaf fiber-reinforced polypropylene hybrid composites. Polym. Compos..

[17] Bledzki A.K., Gassan J. (1999). Composites reinforced with cellulose based fibres. Prog. Polym. Sci..

[18] Huda M.S., Drzal L.T., Mohanty A.K., Misra M. (2006). Chopped glass and recycled newspaper as reinforcement fibers in injection molded poly(lactic acid) (PLA) composites: A comparative study. Compos. Sci. Technol..

[19] Ramakrishnan T., Kumar S.S., Chelladurai S.J.S., Gnanasekaran S., Geetha N.K., Arthanari R., Debtera B. (2022). Effect of Moisture Content on Mechanical Properties of AAM Natural Fiber-Reinforced Isophthalic Polyester Composites. Adv. Mater. Sci. Eng..

[20] Xie Y., Hill C.A.S., Xiao Z., Militz H., Mai C. (2010). Silane coupling agents used for natural fiber/polymer composites: A review. Compos. Part A Appl. Sci. Manuf..

[21] Ramasamy S., Natesan V.T., Balasubramanian K., Justin J.M., Samrot A.V., Jayaraj J.J. (2022). Study on Effect of Fiber Loading Natural *Coccinia Grandis* Fiber Epoxy Composite. J. Nat. Fibers.

[22] Kumar S.S. (2020). Effect of Natural Fiber Loading on Mechanical Properties and Thermal Characteristics of Hybrid Polyester Composites for Industrial and Construction Fields. Fibers Polym..

[23] Al-Oqla F.M., Sapuan S.M. (2014). Natural fiber reinforced polymer composites in industrial applications: Feasibility of date palm fibers for sustainable automotive industry. J. Clean. Prod..

[24] Zhang Y., Li Y., Ma H., Yu T. (2013). Tensile and interfacial properties of unidirectional flax/glass fiber reinforced hybrid composites. Compos. Sci. Technol..

[25] Zhong L.X., Fu S.Y., Zhou X.S., Zhan H.Y. (2011). Effect of surface microfibrillation of sisal fibre on the mechanical properties of sisal/aramid fibre hybrid composites. Compos. Part A Appl. Sci. Manuf..

[26] Raja D.B.P., Retnam B.S.J. (2019). Effect of short fibre orientation on the mechanical characterization of a composite material-hybrid fibre reinforced polymer matrix. Bull. Mater. Sci..

[27] Sreekala M., Kumaran M., Geethakumariamma M., Thomas S. (2004). Environmental effects in oil palm fiber reinforced phenol formaldehyde composites: Studies on thermal, biological, moisture and high energy radiation effects. Adv. Compos. Mater..

[28] Jacob M., Thomas S., Varughese K.T. (2004). Mechanical properties of sisal/oil palm hybrid fiber reinforced natural rubber composites. Compos. Sci. Technol..

[29] Asim M., Saba N., Jawaid M., Nasir M. (2018). Potential of natural fiber/biomass filler-reinforced polymer composites in aerospace applications. Sustainable Composites for Aerospace Applications.

[30] Palomba G., Epasto G., Crupi V. (2022). Lightweight sandwich structures for marine applications: A review. Mech. Adv. Mater. Struct..

[31] Pavlović A., Sintoni D., Minak G., Fragassa C. (2020). On the modal behaviour of ultralight composite sandwich automotive panels. Compos. Struct..

[32] Yanes-Armas S., de Castro J., Keller T. (2017). Long-term design of FRP-PUR web-core sandwich structures in building construction. Compos. Struct..

[33] Oliveira P.R., May M., Panzera T.H., Hiermaier S. (2022). Bio-based/green sandwich structures: A review. Thin-Walled Struct..

[34] Stewart R. (2009). At the core of lightweight composites. Reinf. Plast..

[35] Pradeep S.A., Rodríguez L., Kousaalya A.B., Farahani S., Orrego C., Pilla S. (2022). Effect of silane-treated pine wood fiber (PWF) on thermal and mechanical properties of partially biobased composite foams. Compos. Part C Open Access.

[36] Sternberg J., Pilla S. (2023). Chemical recycling of a lignin-based non-isocyanate polyurethane foam. Nat. Sustain..

[37] Vinson J.R. (2005). Sandwich Structures: Past, Present, and Future. Sandwich Structures 7: Advancing with Sandwich Structures and Materials.

[38] Liu J., Tao J., Li F., Zhao Z. (2020). Flexural properties of a novel foam core sandwich structure reinforced by stiffeners. Constr. Build. Mater..

[39] Shah O.R., Tarfaoui M. (2017). Determination of mode I & II strain energy release rates in composite foam core sandwiches. An experimental study of the composite foam core interfacial fracture resistance. Compos. Part B Eng..

[40] Balıkoğlu F., Arslan N., Demircioğlu T., Inal O., Iren M., Ataş A. (2020). Improving four-point bending performance of marine composite sandwich beams by core modification. J. Compos. Mater..

[41] Farooq U., Ahmad M.S., Rakha S.A., Ali N., Khurram A.A., Subhani T. (2017). Interfacial Mechanical Performance of Composite Honeycomb Sandwich Panels for Aerospace Applications. Arab. J. Sci. Eng..

[42] Du Y., Yan N., Kortschot M.T. (2014). Novel lightweight sandwich-structured bio-fiber-reinforced poly(lactic acid) composites. J. Mater. Sci..

[43] Lascano D., Guillen-Pineda R., Quiles-Carrillo L., Ivorra-Martínez J., Balart R., Montanes N., Boronat T. (2021). Manufacturing and Characterization of Highly Environmentally Friendly Sandwich Composites from Polylactide Cores and Flax-Polylactide Faces. Polymers.

[44] Nickels L. (2017). Car with a biodegradable core. Reinf. Plast..

[45] Galos J., Das R., Sutcliffe M.P., Mouritz A.P. (2022). Review of balsa core sandwich composite structures. Mater. Des..

[46] Mohammadi M.S., Nairn J.A. (2017). Balsa sandwich composite fracture study: Comparison of laminated to solid balsa core materials and debonding from thick balsa core materials. Compos. Part B Eng..

[47] Wu C., Vahedi N., Vassilopoulos A.P., Keller T. (2020). Mechanical properties of a balsa wood veneer structural sandwich core material. Constr. Build. Mater..

[48] Gil L. (2009). Cork Composites: A Review. Materials.

[49] Sousa-Martins J., Kakogiannis D., Coghe F., Reymen B., Teixeira-Dias F. (2013). Behaviour of sandwich structures with cork compound cores subjected to blast waves. Eng. Struct..

[50] Sargianis J., Kim H.-I., Suhr J. (2012). Natural Cork Agglomerate Employed as an Environmentally Friendly Solution for Quiet Sandwich Composites. Sci. Rep..

[51] Hoto R., Furundarena G., Torres J., Muñoz E., Andrés J., García J. (2014). Flexural behavior and water absorption of asymmetrical sandwich composites from natural fibers and cork agglomerate core. Mater. Lett..

[52] Torres J.P., Hoto R., Andrés J., García-Manrique J.A. (2013). Manufacture of Green-Composite Sandwich Structures with Basalt Fiber and Bioepoxy Resin. Adv. Mater. Sci. Eng..

[53] Tondi G., Petutschnigg A. (2016). Tannin-Based Foams: The Innovative Material for Insulation Purposes. Handbook of Composites from Renewable Materials.

[54] Jiang T., Duan Q., Zhu J., Liu H., Yu L. (2020). Starch-based biodegradable materials: Challenges and opportunities. Adv. Ind. Eng. Polym. Res..

[55] Bocz K., Ronkay F., Molnár B., Vadas D., Gyürkés M., Gere D., Marosi G., Czigany T. (2021). Recycled PET foaming: Supercritical carbon dioxide assisted extrusion with real-time quality monitoring. Adv. Ind. Eng. Polym. Res..

[56] Liu X., Li Y., Xing X., Zhang G., Jing X. (2021). Fully recyclable and high performance phenolic resin based on dynamic urethane bonds and its application in self-repairable composites. Polymer.

[57] Taynton P., Yu K., Shoemaker R.K., Jin Y., Qi H.J., Zhang W. (2014). Heat- or Water-Driven Malleability in a Highly Recyclable Covalent Network Polymer. Adv. Mater..

[58] Taynton P., Ni H., Zhu C., Yu K., Loob S., Jin Y., Qi H.J., Zhang W. (2016). Repairable Woven Carbon Fiber Composites with Full Recyclability Enabled by Malleable Polyimine Networks. Adv. Mater..

[59] Home—Mallinda Inc. https://mallinda.com/.

[60] He X., Shi X., Chung C., Lei Z., Zhang W., Yu K. (2021). A sustainable manufacturing method of thermoset composites based on covalent adaptable network polymers. Compos. Part B Eng..

[61] Zhang Y., Yuan L., Liang G., Gu A. (2018). Developing Reversible Self-Healing and Malleable Epoxy Resins with High Performance and Fast Recycling through Building Cross-Linked Network with New Disulfide-Containing Hardener. Ind. Eng. Chem. Res..

[62] de Luzuriaga A.R., Martin R., Markaide N., Rekondo A., Cabañero G., Rodríguez J., Odriozola I. (2016). Epoxy resin with exchangeable disulfide crosslinks to obtain reprocessable, repairable and recyclable fiber-reinforced thermoset composites. Mater. Horiz..

[63] Li X., Zhang J., Zhang L., de Luzuriaga A.R., Rekondo A., Wang D.-Y. (2021). Recyclable flame-retardant epoxy composites based on disulfide bonds: Flammability and recyclability. Compos. Commun..

[64] de Luzuriaga A.R., Markaide N., Salaberria A.M., Azcune I., Rekondo A., Grande H.J. (2022). Aero Grade Epoxy Vitrimer towards Commercialization. Polymers.

[65] La Rosa A.D., Blanco I., Banatao D.R., Pastine S.J., Björklund A., Cicala G. (2018). Innovative Chemical Process for Recycling Thermosets Cured with Recyclamines^®^ by Converting Bio-Epoxy Composites in Reusable Thermoplastic—An LCA Study. Materials.

[66] Ferrari F., Corcione C.E., Striani R., Saitta L., Cicala G., Greco A. (2021). Fully Recyclable Bio-Based Epoxy Formulations Using Epoxidized Precursors from Waste Flour: Thermal and Mechanical Characterization. Polymers.

[67] Cicala G., Pergolizzi E., Piscopo F., Carbone D., Recca G. (2018). Hybrid composites manufactured by resin infusion with a fully recyclable bioepoxy resin. Compos. Part B Eng..

[68] Elium® Resin: A Breakthrough Innovation in Composite Materials|Arkema Global. https://www.arkema.com/global/en/resources/post/elium-resin-breakthrough-innovation/.

[69] Gebhardt M., Chakraborty S., Manolakis I., Meiners D. (2020). Closed-loop room temperature recycling of Elium CFRPs and its influence on the 2nd generation composite properties. J. Plast. Technol..

[70] Carnicero R., Cano L., A Lopez-Manchado M., Verdejo R. (2022). Manufacturing, Testing and Recycling of a small recyclable wind turbine blade. J. Phys. Conf. Ser..

[71] Products & Services—Cecence. https://cecence.com/products-services/.

[72] Andrew J.J., Dhakal H.N. (2022). Sustainable biobased composites for advanced applications: Recent trends and future opportunities—A critical review. Compos. Part C.

[73] Biocomposites Market—Regional & Country Analysis (2022–2029). https://greyviews.com/reports/biocomposites-market/59.

